# Stem and progenitor cell division kinetics during postnatal mouse mammary gland development

**DOI:** 10.1038/ncomms9487

**Published:** 2015-10-29

**Authors:** Rajshekhar R. Giraddi, Mona Shehata, Mercedes Gallardo, Maria A. Blasco, Benjamin D. Simons, John Stingl

**Affiliations:** 1 Cancer Research UK Cambridge Institute, Li Ka Shing Centre, University of Cambridge, Robinson Way, Cambridge CB2 0RE, UK; 2 Telomeres and Telomerase Group, Molecular Oncology Program, Spanish National Cancer Research Centre (CNIO), Melchor Fernández Almagro 3, Madrid E-28029, Spain; 3 Cavendish Laboratory, University of Cambridge, 19 JJ Thomson Avenue Cambridge CB3 0HE, UK; 4 The Wellcome Trust/Cancer Research UK Gurdon Institute, University of Cambridge, Tennis Court Road, Cambridge CB2 1QN, UK; 5 Wellcome Trust-Medical Research Council Stem Cell Institute, University of Cambridge, Tennis Court Road CB2 1QR, UK

## Abstract

The cycling properties of mammary stem and progenitor cells is not well understood. To determine the division properties of these cells, we administered synthetic nucleosides for varying periods of time to mice at different stages of postnatal development and monitored the rate of uptake of these nucleosides in the different mammary cell compartments. Here we show that most cell division in the adult virgin gland is restricted to the oestrogen receptor-expressing luminal cell lineage. Our data also demonstrate that the oestrogen receptor-expressing, milk and basal cell subpopulations have telomere lengths and cell division kinetics that are not compatible with these cells being hierarchically organized; instead, our data indicate that in the adult homeostatic gland, each cell type is largely maintained by its own restricted progenitors. We also observe that transplantable stem cells are largely quiescent during oestrus, but are cycling during dioestrus when progesterone levels are high.

## Abstract (web summary)

The stem and progenitor populations that regulate mammary gland development are debated. Giraddi *et al.* use experimental and mathematical approaches to show that the three lineages of the mammary gland are maintained by their own restricted progenitors, and that cycling status links to the oestrus cycle.

## Graphical abstract

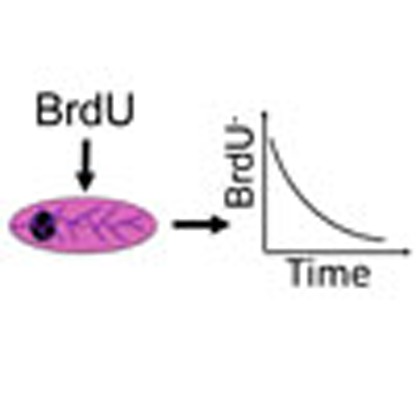

The mouse mammary epithelium is composed of two general lineages of cells, luminal and basal, which are arranged to form a series of ducts that drain alveoli during lactation. The nature of the stem cell(s) that maintain this epithelium is controversial. Initial transplantation experiments using purified cell subsets demonstrated that only the basal cells had the potential to regenerate ductal–lobular outgrowths *in vivo*^1^,^2^,^3^ and are termed mammary repopulating units, or MRUs. Subsequent lineage-tracing studies using luminal- and basal-specific keratin promoters have demonstrated that, in the postnatal mammary gland, the luminal and basal cell compartments are maintained by their own lineage-restricted stem cells^4^,^5^. These findings have been challenged by recent lineage-tracing publications reporting that the mammary epithelium is maintained solely by bipotent basal mammary stem cells, and that the putative luminal stem cells are merely progenitors that are not long lived^6^,^7^.

The nature of putative luminal stem cells, if they exist, is not known. The luminal cell compartment of the mammary epithelium in the non-pregnant mouse is composed of at least three cell types^3^,^8^,^9^. The largest of these has a Sca1^+^CD49b^−^ phenotype and is devoid of any colony-forming potential *in vitro* or engrafting capacity *in vivo*, and thus these cells are termed non-clonogenic luminal (NCL) cells. Most (∼80%) of these cells express the oestrogen receptor (ER) and other markers of luminal differentiation, and are perceived to be differentiated cells^3^,^8^. Alongside NCLs, two further populations of *in vitro* colony-forming cells (CFCs) in the luminal compartment can also be detected: Sca1^−^CD49b^+^ luminal progenitors (termed Sca1^−^ progenitors) that express low levels of luminal cell differentiation markers and Sca1^+^CD49b^+^ luminal progenitors (termed Sca1^+^ progenitors) that express high levels of luminal cell differentiation markers^3^,^8^,^9^,^10^. Analogous luminal cell subpopulations have also been identified in the human mammary epithelium, as EpCAM^+^CD49f^−^ NCL cells, ALDH^+^EpCAM^+^CD49f^+^ luminal progenitors that express low levels of luminal cell differentiation and ALDH^−^EpCAM^+^CD49f^+^ luminal progenitors that express high levels of luminal cell differentiation have all been described^8^,^11^,^12^,^13^,^14^. It is currently not known whether these different luminal cell populations are hierarchically organized.

The focus of the current study is to determine the cell division kinetics of the different mammary epithelial cell subpopulations during mammary gland development, and to use this information to infer the hierarchical relationships between them. Our results demonstrate that most cell division in the adult homeostatic epithelium is localized to the NCL compartment, a cell population currently perceived as being terminally differentiated. Further, our data indicate that the basal, Sca1^−^ progenitors and NCL cells have cell division kinetics that are compatible with each of these subpopulations being largely maintained by their own lineage-restricted progenitors.

## Results

### Cell division during postnatal mammary gland development

**Figure 1:**
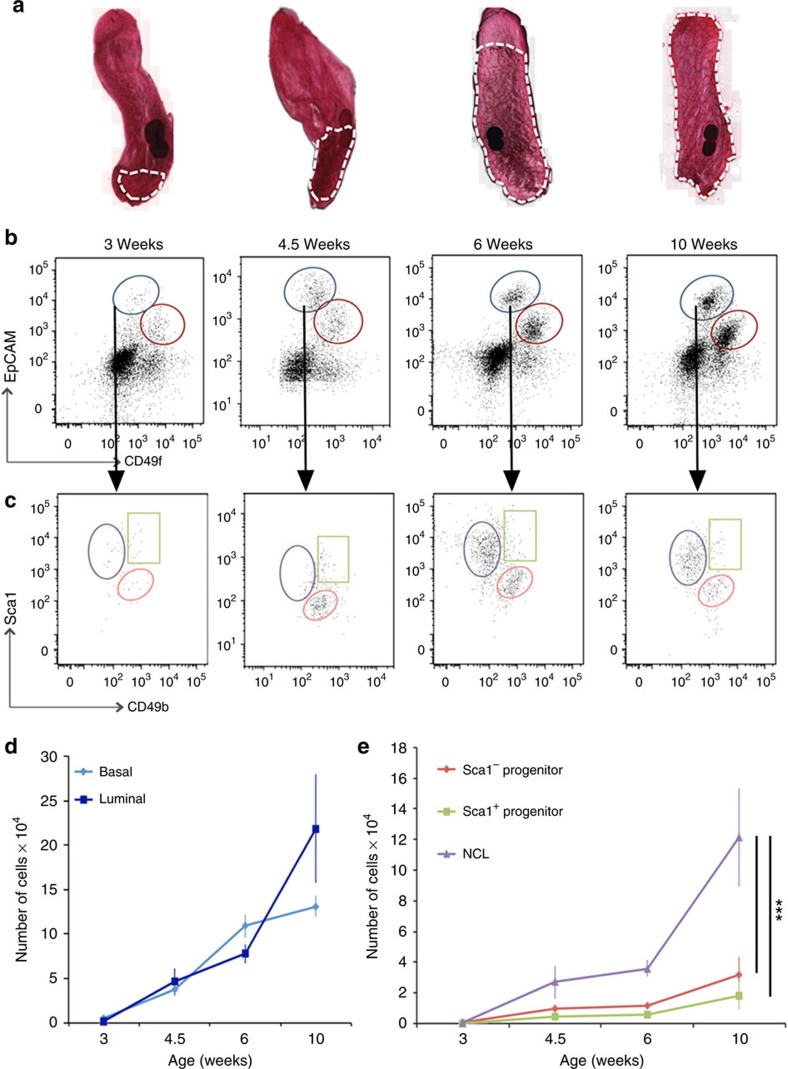
Mammary epithelial cell population changes during postnatal development. (**a**) Representative examples of whole mount carmine alum staining of mammary glands collected at 3, 4.5, 6 and 10 weeks of age in virgin C57Bl6/J mice. White dashes outline the epithelial content. (**b**,**c**) Representative flow cytometry analysis of mammary epithelial cells throughout development. (**b**) Expression of EpCAM and CD49f in live, lin^−^ populations. Dot plots showing the luminal (blue) and basal (red) epithelial compartments. (**c**) Gating strategy of luminal cells, from B, shows further subdivision using Sca1 and CD49b expression. Three populations can be detected: Sca1^+^CD49b^−^ (purple), Sca1^+^CD49b^+^ (green) and Sca1^−^CD49b^+^ (red). (**d**,**e**) Absolute number of epithelial cell subpopulations in the inguinal mammary glands of 3- to 10-week-old C57Bl6/J mice. Mean (±s.e.m) of four independent experiments for each developmental stage for data presented in **a**–**e**. ***P<0.001, as determined by analysis of variance followed by a Dunnett’s multiple comparison test.

Our first objective was to identify which cell types are dividing during postnatal mammary gland development. To this end, we first investigated how the sizes of the different subpopulations change during development. Mammary cells isolated from 3–week-old (pre-pubertal), 4.5- and 6-week-old (pubertal) and 10-week-old (adult) C57Bl6/J mice were stained to detect epithelial cell adhesion molecule (EpCAM), CD49f, Sca1 and CD49b, and analysed using flow cytometry to determine the number of the basal and luminal cells among the lineage^−^ (CD31^−^, CD45^−^ and Ter119^−^) cell subpopulations (Fig. 1a-c; a representative image showing the mammary epithelial gating strategy for all sorting experiments in this study is shown in Supplementary Fig. 1). As expected, the absolute number of basal and luminal cells increases dramatically between 3 and 10 weeks of age (Fig. 1d and Supplementary Table 1A). However, within the luminal compartment, the NCL cell subpopulation displayed the greatest increase in cell number during the 3- to 10-week developmental period (Fig. 1e). When the gland reaches the mature virgin state at 10 weeks of age, the basal, Sca1^−^ progenitors, Sca1^+^ progenitors and NCL cells comprise ∼37%, 9%, 5% and 34% of the total mammary epithelium, respectively (Supplementary Table 1A); the remaining cells are cells with an indeterminate phenotype. The expansion of the luminal progenitor populations and basal MRUs throughout pubertal development was confirmed using CFC^8^ and MRU assays, respectively (Supplementary Table 1B).

**Figure 2:**
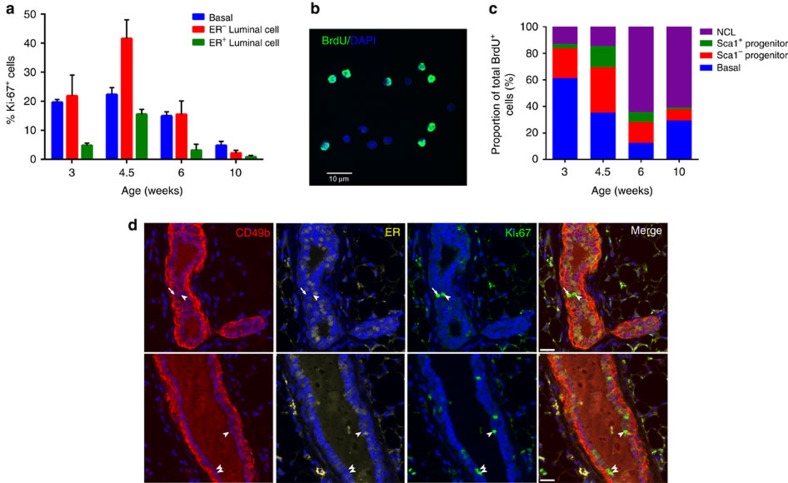
Cell division in the mammary gland during postnatal development. (**a**) Quantification of Ki-67 staining among basal, ER^−^ luminal and ER^+^ luminal cells in intact mammary glands of 3- to 10-week-old C57Bl6/J mice. Mean±s.e.m of four independent samples for each developmental stage. Between 10 and 35 mammary ducts were scored for each sample in the different developmental stages. (**b**) Representative image of sorted mammary epithelial cells stained by immunofluorescence for BrdU. Scale bar, 10 μm. A total of 12 independent samples were analysed. (**c**) Distribution of different mammary epithelial cell subpopulations that incorporated BrdU in the inguinal mammary glands of 3- to 10-week-old C57Bl6/J mice. Mean±s.e.m of three independent samples for each developmental stage. (**d**) Immunostaining of representative mammary glands from adult (≥10 weeks) C57Bl6/J mice showing the presence of Ki-67^+^ER^−^CD49b^−^ NCL cells (arrowheads) and Ki-67^+^ER^−^CD49b^+^ progenitors (arrow). Scale bars, 20 μm. A total of four independent mice were analysed.

To determine the proportion of the different mammary cell types that are proliferating at different stages of development, glands from 3-, 4.5-, 6- and 10-week-old mice were sectioned and immunostained to detect keratin 5 (K5), ER and Ki-67. Results indicate that, as development progresses, the proportion of proliferating cells among the basal, luminal ER^−^ and luminal ER^+^ subpopulations decreases (Fig. 2a). When we discriminated between cell proliferation in the terminal end buds versus the ducts in 3- to 6-week-old mice, we observe that the relative levels of cell proliferation between the basal, ER^−^ luminal cells and ER^+^ luminal cells is similar in both structures, but that the terminal end buds on average have much higher levels of cell division (Supplementary Fig. 2A,B). Consistent with a previous report^15^, the highest proportion of proliferating cells at all stages of development are the basal and ER^−^ luminal cells. Although 3%–16% of ER^+^ cells are Ki-67^+^ during puberty, only 0.8±0.5% of ER^+^ cells in the adult gland are Ki-67^+^, an observation that is consistent with previous reports^16^,^17^.

One drawback of examining cell proliferation on tissue sections is that it is currently not possible to consistently resolve the Sca1^−^ and Sca1^+^ progenitor populations from the NCL cells. To circumvent this difficulty, 3-, 4.5-, 6- and 10-week-old mice were injected with 5-bromodeoxyuridine (BrdU) and 6–7 h later (the approximate duration of S-phase^18^ and Supplementary Table 2) the mammary cells were harvested, dissociated and the basal, Sca1^−^ luminal progenitors, Sca1^+^ luminal progenitors and NCL cells were purified as shown in Fig. 1b,c. The percentage of BrdU^+^ cells in each sorted subpopulation was enumerated by fluorescence microscopy (Fig. 2b) and this information, after adjusting for population size, was then used to determine the absolute number of BrdU^+^ cells in each cell subpopulation in a pair of inguinal mammary glands (Fig. 2c and Table 1). This analysis demonstrates that most cell division in early puberty (3–4.5 weeks) occurs in Sca1^−^ luminal progenitors and basal cells; however, as mice mature into adulthood, most of the cell proliferation becomes concentrated within the NCL cell compartment. In fact, the number of BrdU^+^ NCL cells increases ∼24-fold from 3 to 10 weeks (Table 1), whereas the number of BrdU^+^ basal, Sca1^−^ progenitors and Sca1^+^ progenitors increases only up to 2-fold during this same time period. In the 10-week-old mouse, ∼62% of all the BrdU^+^ cells that are detected are within the NCL cell subpopulation (Fig. 2c), an observation that is highly unexpected considering that these cells are deemed to be non-clonogenic. The observed number of BrdU^+^ NCL cells far exceeds the proliferative output generated by the luminal progenitor compartment, as 1,500 BrdU^+^ Sca1^−^ and Sca1^+^ luminal progenitors cannot generate 9,500±3,600 BrdU^+^ NCL cells during the 7-h BrdU chase period of this experiment (Supplementary Table 3A), indicating that the majority of these BrdU^+^ NCL cells are not the immediate daughters of luminal progenitor cells. Based on this, we conclude that most cell division in the non-pregnant adult mammary gland is restricted to the NCL subpopulation, and that these cells can generate NCL progeny. A similar conclusion is obtained in a separate experiment where mice were injected twice, 6 h apart, with the synthetic nucleoside 5-chloro-2′-deoxyuridine (CldU) and the different cell subpopulations analysed for CldU uptake. We observed that in six of the eight mice analysed, the number of CldU^+^ NCL cells greatly exceeds (up to sevenfold) the number of CldU^+^ luminal progenitors (Supplementary Table 3B). Interestingly, most of this cell division in the NCL subpopulation must occur in the ER^−^ subset, as <1% of ER^+^ luminal cells in adult mice express Ki-67 (Fig. 2a). The presence of these proliferating ER^−^ NCL cells in intact tissue was confirmed by immunostaining mammary glands from adult mice to detect Ki-67^+^CD49b^−^ NCL cells (Fig. 2d and Supplementary Fig. 2C).

### Cell division during the oestrus cycle

**Figure 3:**
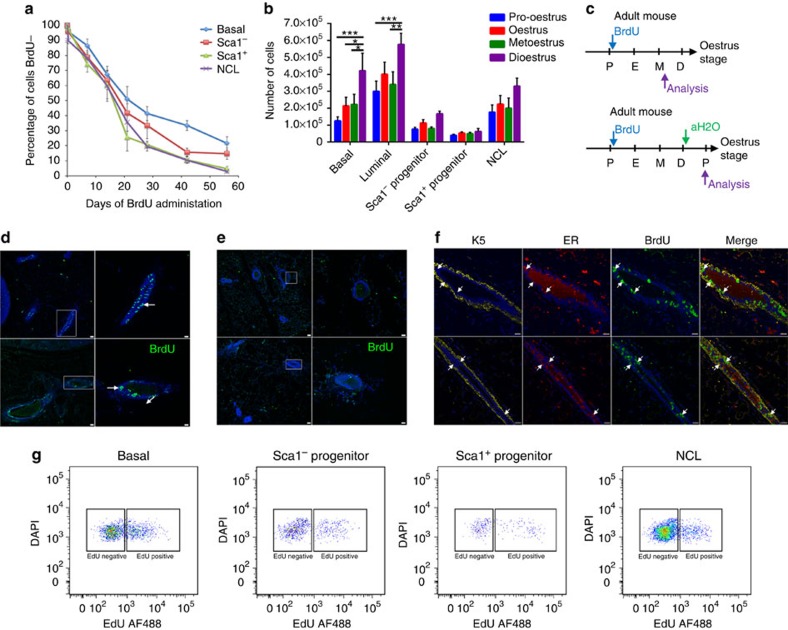
Epithelial cell turnover in the adult mammary gland. (**a**) Loss of BrdU^−^ cells over time among basal (blue), Sca1^−^ progenitor (red), Sca1^+^ progenitor (green) and NCL (purple) subpopulations. BrdU was administered continuously to 24 adult, 10-week-old mice for up to 8 weeks, and 3 mice were analysed at each timepoint. (**b**) Absolute number of different mammary epithelial cell subpopulations in the inguinal mammary glands of adult (≥10 weeks) C57Bl6/J mice in different stages of the oestrus cycle. Mean±s.e.m of five independent mice per oestrus stage. ***P<0.001, **P<0.01, *P<0.05 as determined by analysis of variance followed by a Tukey’s multiple comparisons test. (**c**) Protocol: adult (≥10-week-old) mice in pro-oestrus were treated with BrdU continuously (blue arrow) before culling for analysis (purple arrow), or until dioestrus where BrdU was replaced with water (green arrow) until the following pro-oestrus where mice were analysed (purple arrow). (**d**) Staining of BrdU-treated mammary glands. Arrows indicate clusters of BrdU^+^ cells. Representative images from a pool of total four independent samples. Scale bars, 30 μm. The right panels in **c** are close-ups of the respective areas marked with a white rectangle. Scale bars, 16 μm. (**e**) Representative images of BrdU staining in mammary glands of mice treated with BrdU:water during one complete oestrus cycle. Very few BrdU^+^ cells were detected. A total of four independent mice were analysed. Scale bars, 30 μm, right panels are close-ups of rectangle. Scale bars, 16 μm. (**f**) Immunostaining of representative mammary glands from adult (≥10-week-old) mice treated continuously with BrdU, showing BrdU^+^ER^+^ cells (arrows), and stained with K5 to indicate the basal compartment. Scale bars, 20 μm. A total of four independent mice were analysed. (**g**) Representative flow cytometry analysis of adult (≥10-week-old) C57Bl6/J mice treated continuously with EdU from pro-oestrus to metoestrus. A total of four independent mice were analysed.

The data presented above demonstrate that in the non-pregnant adult mammary gland, most cell division is restricted to the NCL subpopulation. To determine how often these and other cell subtypes enter the cell cycle, we administered BrdU continuously via the drinking water to 10-week-old mice for varying chase times up to 8 weeks and assessed the proportion of cells that remained BrdU^−^ over time. We observed that the decline in the frequency of BrdU^−^ cells follows an approximately exponential decay pattern from 100% at the start of the experiment towards 0% at 8 weeks (Fig. 3a). Such exponential time dependence is consistent with separate compartments being defined by a lineage-restricted progenitor cell population, in which cells exit from quiescence and enter sporadically into the cell cycle (see Methods). If we assume (see following section) that the majority of cells have proliferative potential, then the time between consecutive entries into cell cycle (*T*_C_) for the different cell subpopulations can be derived from these BrdU decay curves and are estimated to be 35±4, 26±3, 18±3 and 21±3 days for basal, Sca1^−^ progenitors, Sca1^+^ progenitors and NCL cells, respectively (Table 2 and Methods).

The exponential character of the decay of BrdU^−^ cells in the long term suggests that the exit from quiescence occurs stochastically, with the time between consecutive events being statistically uncorrelated (that is, Markovian). However, once activated, these progenitors must divide rapidly to account for the observed expansion of cell numbers during the oestrus cycle (Fig. 3b), before undergoing regression following dioestrus. As a confirmation to detect these highly proliferative cells within a single oestrus cycle, BrdU was administered continuously to mice via the drinking water as they progressed from the early (pro-oestrus/early oestrus) stages of the oestrus cycle to a late stage (metoestrus) 2–3 days later (Fig. 3c). As shown in Fig. 3d, abundant clusters of BrdU^+^ cells can be identified, with many of these cells also expressing ER (Fig. 3f), thereby indicating a high degree of cell turnover within the NCL compartment, which results in the generation of ER^+^ progeny. However, these clusters of BrdU^+^ cells were mostly absent when the mice were permitted to progress through dioestrus and analysed at the following pro-oestrus (Fig. 3e), thereby indicating that many of the cells that proliferated during the oestrus cycle underwent apoptosis at the end of the cycle.

To further identify which cells undergo cell division within a single oestrus cycle, adult mice were administered 5-ethynyl-2'-deoxyuridine (EdU) continuously via the drinking water as they progressed from pro-oestrus to metoestrus, and when they got to metoestrus the glands were analysed by flow cytometry to identify EdU^+^ cells. Our results indicate that 19±5%, 15±5%, 29±9% and 13±2% of basal, Sca1^−^ progenitors, Sca1^+^ progenitors and NCL cells were EdU^+^, respectively (Fig. 3g and Supplementary Table 4A). However, when these values were normalized to take in account the sizes of the different cell subpopulations, it is observed that most cell division within the mammary epithelium during the course of a single oestrus cycle is localized to the NCL compartment, as 64±7% of all EdU^+^ cells were within this subpopulation (Supplementary Table 4A).

**Figure 4:**
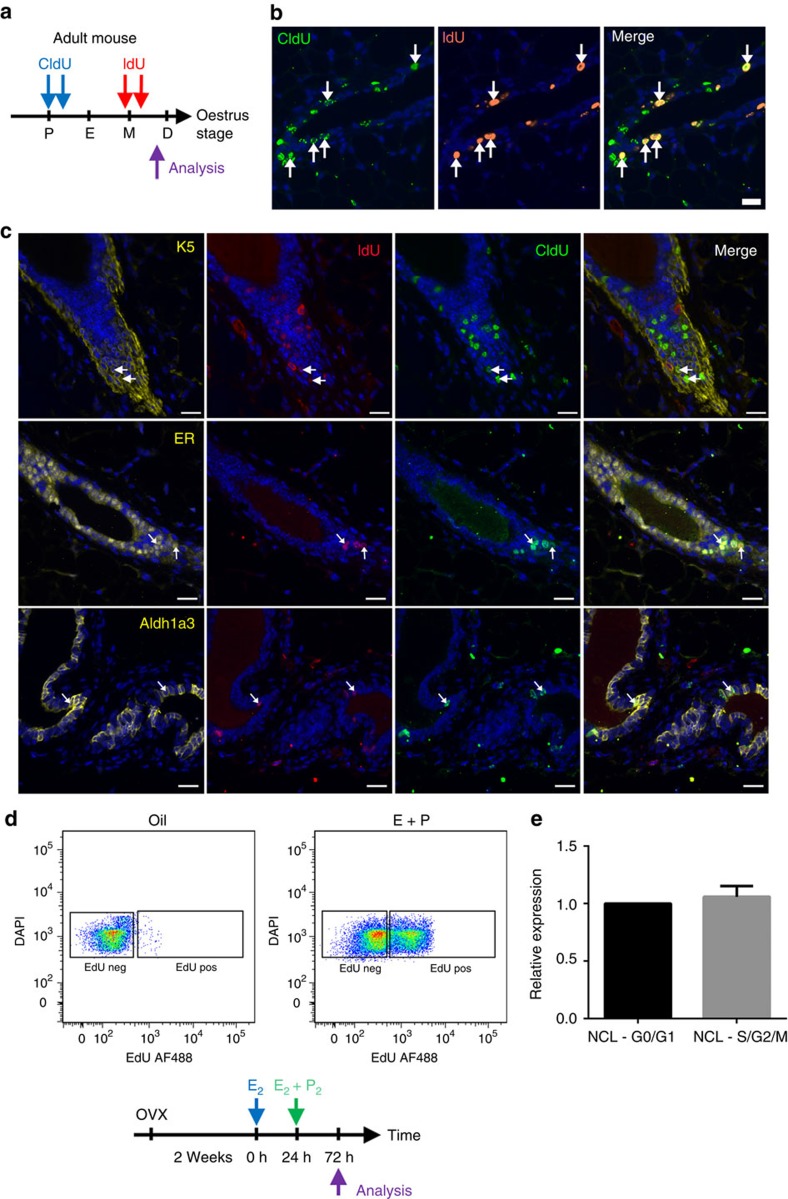
Cell proliferation during the oestrus cycle. (**a**) Protocol: adult (≥10-week-old) C57Bl6/J mice were administered CldU during pro-oestrus/early oestrus (blue arrows) and then IdU at metoestrus (red arrows) to detect sequential proliferation within the mammary gland. Immunofluorescent staining of adult mammary glands stained with CldU and IdU (**b**), and with ER, Aldh1a3 or K5 (**c**). Arrows indicate CldU^+^IdU^+^ cells, which demonstrate sequential cell division among mammary epithelial cells during the oestrus cycle. Representative image from four independent samples. Scale bars, 20 μm. (**d**) Representative flow cytometry dot plot showing the incorporation of EdU by NCL cells following injection of either oestrogen and progesterone or oil. A total of six independent adult (≥10-week-old) mice were analysed (four injected with oestrogen and progesterone, two injected with oil only). (**e**) NCL cells from adult (≥10-week-old) mice that are either in G0/G1 versus S/G2/M stages of the cell cycle were analysed by quantitative RT–PCR for ERα mRNA expression relative to Gapdh mRNA. Expression is relative to that of NCL G0/G1 cells. Mean±s.e.m of five independent mice.

To identify the cell types that are capable of repeated cycling during a single oestrus cycle, we administered CldU to mice that were in the early stages of the oestrus cycle (pro-oestrus/oestrus) followed by administration of IdU during the latter part of the oestrus cycle (metoestrus; Fig. 4a,b). Glands from these mice were then immunostained to detect CldU, IdU and either a marker of NCL cells (ER), Sca1^−^ luminal progenitors (Aldh1a3) or basal cells (K5; Fig. 4c). Our results demonstrate that the NCL cells had the highest rate of cell cycle re-entry, as 67±12% of CldU^+^IdU^+^ cells were also ER^+^ (Fig. 4c). Basal cells had the second highest level of cell cycle re-entry, as 31±7% of CldU^+^IdU^+^ cells were K5^+^, whereas only 17±5% of CldU^+^IdU^+^ were Aldh1a3^+^. This data fits well with the data presented in Fig. 3b showing that the number of NCL and basal cells increase the most during a single oestrus cycle. Collectively, the low rate of BrdU incorporation combined with the dramatic increase in the size of the tissue through the oestrus cycle suggests that, in any given oestrus cycle, proliferation is restricted to a minority of progenitors, which are mostly NCL cells and, to a lesser degree, basal cells, that undergo a burst of proliferation to generate progeny that subsequently undergo apoptosis at the end of the oestrus cycle.

### Each lineage is maintained by its own progenitors

To interpret the 8-week BrdU incorporation data presented above, we made the important assumption that the separate compartments comprised predominantly of progenitors with the majority of cells capable of cell division. Consistent with this hypothesis, we have previously reported that basal cells, Sca1^−^ progenitors and Sca1^+^ progenitors all have cloning efficiencies between 25% and 40% when seeded into culture^5^,^8^. Considering that flow-sorting toxicity is estimated to be in the range of 50%–75% (refs 5, 8, 19), this indicates that most, and perhaps all, of these cells have the potential to enter the cell cycle.

However, the potential of NCL cells to enter the cell cycle *in vivo* is not well characterized. To investigate this further, 10-week-old mice were ovariectomized and 2 weeks later they were injected with 10 μg of oestrogen, to upregulate the progesterone receptor^20^ but not to induce cell proliferation^21^ (Supplementary Fig. 3A,B), and after injection they were continuously administered EdU. After 24 h, the mice were given another injection containing 10 μg oestrogen and 1 mg progesterone, to induce cell proliferation. After 48 h, the glands were removed and the frequency of EdU^+^ cells among the different mammary epithelial cell subpopulations was determined using flow cytometry. Our results indicate that 46±6% of all NCL cells were EdU^+^ (Fig. 4d), and, after adjusting for population size, that 67±3% of all EdU^+^ cells in the mammary epithelium were localized in the NCL subpopulation (Supplementary Table 4B). Our results indicate that the basal compartment is also highly responsive to hormonal stimuli with ∼27% of basal cells being EdU^+^ (Supplementary Table 4B). This indicates that the NCL population and, to a lesser degree, the basal population are highly responsive to the mitogenic effects of oestrogen and progesterone, and that a large fraction of these cells can be recruited into the cell cycle within a short time period.

This high degree of responsiveness of the NCL cells to these hormones *in vivo* is somewhat counter-intuitive, considering that this population is composed predominantly of ER^+^ cells^8^, and ER^+^ cells rarely express Ki67 in the adult mammary epithelium. A previous study, in which oestrogen was administered to ovariectomized mice and how this influenced ERα expression was examined, concluded that non-cycling ERα^+^ cells are not terminally differentiated, but instead these cells have proliferative potential, as it was demonstrated that ERα is downregulated on recruitment into the cell cycle in response to hormone treatment, and that ERα protein expression is re-established following cell division^22^. To investigate this phenomenon further, we isolated NCL cells at different stages of the cell cycle (G_0_/G_1_ versus S/G_2_/M) via incorporation of Hoechst 33342 into the DNA of viable cells and then used quantitative reverse transcription–PCR (RT–PCR) to measure ERα messenger RNA levels in these cells. Our results indicate that cycling and non-cycling NCL cells have similar amounts of ERα transcripts (Fig. 4e), which indicates that cycling ER^−^ NCL cells are not a distinct undifferentiated precursor cells for the ER^+^ cell lineage; instead, these results are consistent with the model that ER protein expression merely fluctuates with the cell cycle. Collectively, this data demonstrates that a significant fraction of NCL cells have the potential to be recruited into the cell cycle when exposed to ovarian steroid hormones.

To further challenge the hypothesis that the separate epithelial populations comprise predominantly of progenitors, we combined the long-term data with short-term CldU incorporation. Specifically, we injected eight adult mice with CldU at two times separated by 6 h and determined the proportion of CldU^+^ cells in the different mammary cell subpopulations. Results demonstrate that 4.7±0.5%, 6.3±0.7%, 3.0±0.3% and 8.8±0.5% of basal cells, Sca1^−^ luminal progenitors, Sca1^+^ luminal progenitors and NCL cells incorporated the nucleoside, respectively. Then, if all cells have indeed had an equal chance of entering into the cycle, with an S-phase length (*T*_S_) of ∼7 h, we expect a fraction of around 2 × (6+2*T*_S_) h/*T*_C_ of cells to take up CldU following the double injection, where *T*_C_ denotes the average timescale for entry into cell cycle, and the factor of 2 accounts for cell doubling following mitosis (see Methods). From the measured fractions, this translates to cell cycle entry times of around 35±4, 26±3, 55±6 and 19±1 days for basal, Sca1^−^ progenitors, Sca1^+^ progenitors and NCL cells, respectively. With the exception of Sca1^+^ progenitors, these figures are remarkably consistent with that found from the long-term study of continuous BrdU incorporation (Table 2), suggesting that these three compartments comprise a pure progenitor population. As continuous long-term exposure to BrdU measures cell division and net migration of BrdU^+^ cells between the different cell compartments, and short-term exposure to CldU measures cell division within the different compartments, then the observation that identical timescales of cell cycle re-entry are calculated for the two methods indicates that there is no significant migration of BrdU-labelled NCL, Sca1^−^ luminal progenitors and basal cells between compartments in adult mice during the 8-week duration of the assay, and thus they are largely maintained by their own lineage-restricted progenitors. It should be noted that this conclusion relies on the assumption that each of these subpopulations is composed predominantly of progenitors with the majority of cells being able to enter the cell cycle.

In our analysis above, only the timescale for the minority Sca1^+^ progenitor population shows significant discrepancy (18 days versus 55 days). To explain this discrepancy, we considered how the results would be modified if we considered a two-compartment model in which the Sca1^+^ population included a subset of cells that had terminally differentiated. In this case, the fraction of BrdU+ cells would be proportionately adjusted in both the short- and long-term assay. (For details of how the BrdU^+^ cell fractions are modified, refer to Methods.) Indeed, from a least-squares fit of the predicted time dependences to the data, we find that the short- and long-term data can be rationalized with a cell cycle entry time, *T*_C_, of 28±10 days with some 50% of cells in the Sca1^+^ compartment terminally differentiated.

### Mammary cell populations have disparate telomere lengths

**Figure 5:**
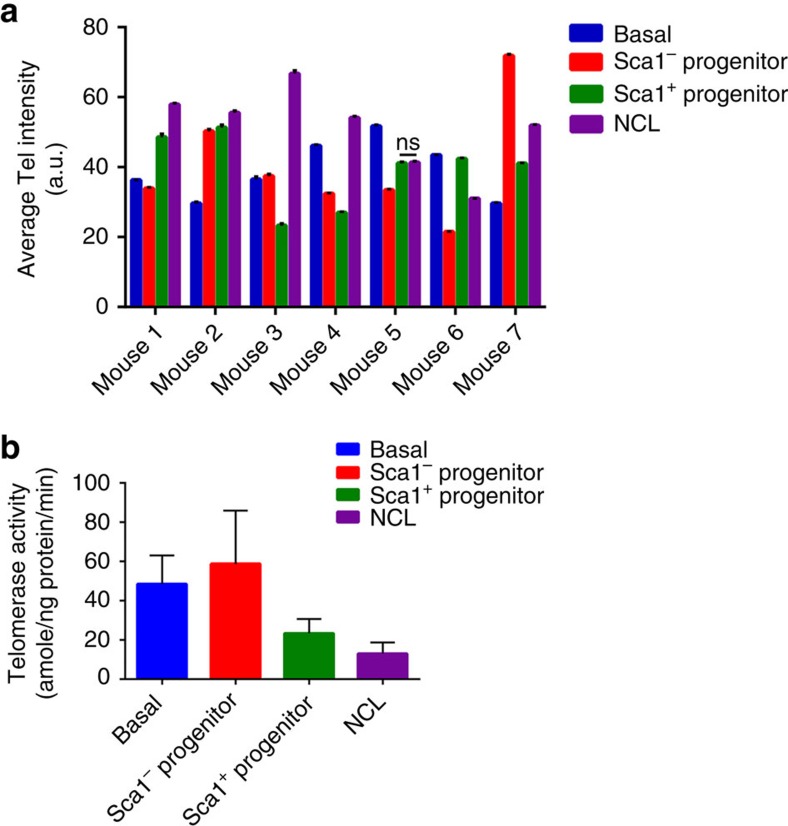
Telomere length and telomerase activity among mammary epithelial cell subpopulations. (**a**) Average telomere (Tel) intensity (a.u.) of the different sorted mammary epithelial cell populations in seven different adult (≥10-week-old) mice. Between 1,200 and 8,100 telometric spots were counted for each subpopulation. Error bars indicate s.d. All subpopulations within individual mice have telomere lengths that are significantly (***P<0.001, analysis of variance (ANOVA) followed by a Dunnett’s multiple comparison test) different from telomere lengths of NCL cells, except where indicated (ns). (**b**) Quantification of telomerase activity in adult (≥10-week-old) C57Bl6/J mice in the different sorted populations. Telomerase activity is shown as amole of product min^−1^ ng^−1^ total cellular protein. Error bars indicate s.e.m. A total of seven independent experiments were analysed. A one-way ANOVA followed by a Dunnett’s multiple comparison test was carried out and no statistical differences were observed.

To further confirm the compartmentalization of the different mammary epithelial cell subpopulations, we purified the four different mammary epithelial cell types from seven individual mice and measured their mean telomere length using quantitative fluorescence *in situ* hybridization^23^. If the NCL cells were the progeny of either of the luminal progenitor populations, then one would predict that the NCL cells should, on average, have shorter telomeres than their perceived parents, as telomeres, in the absence of telomerase, erode with successive rounds of proliferation. However, as shown in Fig. 5a, we observe no correlation between cell subtype and telomere length. For example, in four of the seven mice examined, the NCL population had longer telomeres than their corresponding Sca1^+^ and Sca1^−^ progenitors. These longer telomeres are not due to increased levels of telomerase activity in NCL cells, as we observed that these cells do not have significantly higher telomerase activity than any of the other cell subpopulations (Fig. 5b). Collectively, these results suggest that luminal progenitors do not give rise to NCL progeny, as it would be expected that if these cells are hierarchically organized, then the NCL cells should, on average, have shorter telomeres than their parents, which is not necessarily the case.

### MRU cell division kinetics

Basal and MRU numbers in FVB mice have been previously described to fluctuate 4- and 14-fold, respectively, during the different stages of the oestrus cycle, with maximal numbers of both observed during dioestrus^24^. We observe a similar fluctuation of basal and MRU numbers in C57Bl6/J mice, with up to a threefold increase in basal cell numbers (Fig. 3b) and up to a sevenfold increase in MRUs in dioestrus compared with other oestrus stages (Supplementary Table 5). To investigate the cycling properties of MRUs during the oestrus cycle, we injected the S-phase cytotoxin 5-fluorouracil (5FU) into adult mice that were either in oestrus or in dioestrus and performed MRU assays. If a proportion of MRUs are in S-phase during 5FU exposure, then the total number of detected MRUs/pair of glands should be proportionally lower than the number present in untreated mice. Our results demonstrate that MRUs in mice in oestrus are insensitive to 5FU, as approximately equal numbers (∼5,000 MRUs per pair of glands) of MRUs are present in both untreated and treated mice (Table 3). However, MRUs in mice that were in dioestrus at the time of 5FU administration are highly sensitive to 5FU, as there is a ∼75% reduction in MRU numbers on 5FU exposure. This indicates that MRUs are largely quiescent when progesterone levels are low during oestrus and the majority are cycling when progesterone levels are higher during dioestrus.

## Discussion

The mammary gland undergoes considerable morphological changes during the oestrus cycle^24^,^25^. We observe that in the adult virgin mouse, initial recruitment of mammary epithelial cells into the cell cycle at the beginning of the oestrus cycle is stochastic, but once recruited the cell division is repeated to account for the expansion of cell numbers during the oestrus cycle. At the end of the oestrus cycle, most of the newly generated daughter cells undergo apoptosis and the process repeats itself during the next oestrus cycle^25^. Basal cell proliferation in response to oestrogen and progesterone has been well documented^24^,^26^; however, the observation that the NCL compartment is highly responsive to the mitogenic effects of these hormones and accounts for the largest increase in cell number during the oestrus cycle is novel. Gene expression profiling of normal NCL cells derived from both humans and mice reveals that these cells have a gene expression profile that resembles that obtained from Luminal A and Luminal B tumours^8^,^13^,^14^, which account for ∼50% of all breast cancers. It is currently not known whether any of these NCL cells can serve as a cell-of-origin for ER^+^ breast cancer.

We also observed that, in the non-pregnant homeostatic adult mammary epithelium, the basal, Sca1^−^ luminal progenitor and NCL compartments appear to be largely maintained by their own lineage-restricted committed progenitors, as we fail to detect any meaningful migration of BrdU-labelled cells in or out of these compartments over an 8-week time course. It is currently not known whether the different luminal cell compartments are maintained by their own lineage-restricted committed progenitors during other developmental states such as puberty, pregnancy or in the post-menopausal gland. Owing to the limited sensitivity of our approach, our data cannot rule out very low levels of cell migration between compartments; however, our data are not compatible with a direct stepwise differentiation from basal cells to luminal progenitors, to NCL cells in the adult homeostatic gland. Our analysis is also dependent on a model that assumes that each separate subpopulation of cells is predominantly composed of progenitors that are capable of cell division; our observation that equal cell division timescales are obtained for the basal, Sca1^−^ progenitor and NCL subpopulations for both short- and long-term nucleoside exposures supports this assumption. Our conclusion regarding the compartmentalization of the different mammary cell subpopulations is in excellent agreement with a recent lineage-tracing paper in which Sca1^−^ (Wap-expressing) luminal progenitors were marked during late pregnancy and then followed up through involution for a total of 10 weeks^27^. The marked cells were restricted to only the Sca1^−^ and Sca1^+^ luminal progenitor cell populations, but no marked NCL cells were detected. The lack of marked NCL cells over such a long time period challenges the hypothesis that luminal progenitors give rise to NCL cells.

It has been recently reported that Procr identifies rare bipotent stem cells in the mouse mammary epithelium^7^. When these cells were genetically tagged in 8-week-old virgin mice and their fate tracked over a subsequent 6-week period, it was observed that most of the clones generated from these Procr^+^ cells were composed of ten cells or less. Considering that 6 weeks is approximately eight to nine oestrus cycles, and that each oestrus cycle involves the approximate doubling of the number of epithelial cells within the mammary gland, then this indicates that Procr^+^ cells have a minimal contribution to the day-to-day turnover within the mammary epithelium, and that their contribution to mammary gland homeostasis is only significant over much longer time scales.

In support of our conclusion that the basal, Sca1^−^ luminal progenitors and NCL cells are largely maintained by their own restricted progenitors, we observed that in some mice the telomere length of the NCL cells was longer than the telomere lengths of both the luminal progenitor cell populations, an observation that challenges the perceived parent–progeny relationship between these cells. A similar finding was recently observed in NCL cells derived from human tissue^28^. Interestingly, although NCL cells (referred to as ‘luminal cells (LCs)’ in ref. 28) in the human have long telomeres in breast tissue derived from younger women, the telomere length decreases as the age of the tissue donor increases. This decrease in telomere length in NCL cells with age is consistent with the high level of cell proliferation present in these cells.

In summary, our data indicate that the three lineages of the mammary epithelium (myoepithelial, ER^+^ and milk secreting) are each maintained by their long-lived committed progenitor cells that provide cell regeneration during the day-to-day turnover of the mammary epithelium. It is interesting to note that a similar organization of autonomous cellular compartments, each maintained by its own stem cell, was recently reported for the skin^29^. Considering that the mammary epithelium is a derivative of the skin, such a compartmentalization of stem cells and their progeny is not entirely unexpected.

## Methods

### Whole-mount Carmine staining

The inguinal mammary glands from 3-, 4.5-, 6- and 10-week-old female C57Bl6/J mice (Harlan Laboratories) were fixed overnight in Carnoy’s fixative. Following fixation, the glands were washed with 70% ethanol for 15 min, gradually rinsed in water and stained overnight in Carmine Alum staining solution. Stained glands were washed in 70% ethanol. All animal work in this study was approved by the Cancer Research UK Cambridge Institute Animal Welfare and Ethics Review Body, and was performed under Home Office Animal Licenses 80/2223 and 70/7746.

### Oestrus staging of adult mice

Adult mice (≥10 weeks of age) were given a vaginal flush with 60 μl of sterile PBS at the same time daily. The vaginal smears were then spotted onto a microscope slide and allowed to adhere for 5–10 min. For initial cycle assessment, smears were visualized under a light microscope and stages categorized depending on the presence and/or proportion of nucleated epithelial cells, cornified cells and lymphocytes^30^,^31^. Microscope slides were then haematoxylin and eosin-stained and stages visually confirmed. As mice were oestrus staged daily, we were able to identify mice that got stuck in certain oestrus stages and were considered non-cycling; these mice were not used for experiments.

### Dissociation of mammary tissue into single cell suspensions

Number 4 (inguinal) mammary glands from virgin female C57Bl/6J mice (Harlan Laboratories) were digested overnight in DMEM/F-12 supplemented with 15 mM Hepes (Invitrogen), 1 mg ml^−1^ collagenase A (Roche), 100 U ml^−1^ hyaluronidase (Sigma) and 50 μg ml^−1^ gentamycin (Gibco). No growth factors or serum were included in the digestion mixture, to avoid induction of cell proliferation. After dissociation, the red blood cells were lysed with NH_4_Cl followed by sequential digestion with 0.25% trypsin (StemCell Technologies), 5 mg ml^−1^ dispase (StemCell) and 0.1 mg ml^−1^ DNase (Sigma). The cell preparation was then filtered through a 40-μm mesh (BD Biosciences) to obtain a single cell suspension. Ice-cold Hank’s balanced salt solution (Gibco) supplemented with 2% fetal bovine serum (Gibco) and 10 mM HEPES (Sigma; collectively termed HF) was used for all the washing steps. In some experiments, the mice were oestrus staged as outlined above.

### Preparation of cells for flow cytometry

Single cell suspensions of mammary cells were pre-blocked with 10% normal rat serum diluted in HF for 10 min at 4 °C, followed by incubation with primary antibodies diluted in HF for 10 min at 4 °C. The primary antibodies included 1 μg ml^−1^ CD31-biotin (clone 390, eBioscience), 1 μg ml^−1^ CD45-biotin (clone 30-F11, eBioscience), 1 μg ml^−1^ Ter119-biotin (clone Ter119, eBioscience) and 1 μg ml^−1^ BP-1-biotin (clone 6C3, eBioscience) as lineage markers (for depletion of haematopoietic and endothelial cells), and 1 μg ml^−1^ EpCAM-AlexaFluor 647 (clone G8.8, Biolegend), 2 μg ml^−1^ CD49f-AlexaFluor 488 or CD49f-Pacific Blue (clone GoH3, Biolegend), 1 μg ml^−1^ CD49b-PE (clone HMα2, Biolegend) and 1 μg ml^−1^ Sca-1-PE-Cy7 (clone D7, BioLegend). Matched isotypes with respective fluorochromes were used at the same concentrations to set gates and single stained control cells were used for compensation. After primary antibody incubation, cells were washed with ice-cold HF and incubated with 0.4 μg ml^−1^ streptavidin–allophycocyanin (APC)–Cy7 (BioLegend) for 10 min at 4 °C. 1 μg ml^−1^ 4',6-diamidino-2-phenylindole (DAPI, Invitrogen) was used to discriminate live cells during flow cytometry gating. After the staining protocol, cells were filtered through a 30-μm cell strainer before sorting. Cells were analysed using the BD LSRII or BD FACSAria instrument and the latter was used for sorting experiments. Flow cytometry data were analysed using either FlowJo (version 8.6, Tree Star Inc.) or BD FACSDiva (version 5.0.1).

### Ovariectomy and steroid hormone treatments

Female C57Bl/6J mice, 10–12 weeks old, were bilaterally ovariectomized and allowed to recover for 2 weeks. Mice were injected subcutaneously with 100 μl of sesame oil alone (vehicle) or with 10 μg 17β-estradiol (Sigma). Mice were then given a single intraperitoneal (i.p.) injection of 10 mg ml^−1^ EdU (Life Technologies) in PBS at a dose of 100 mg kg^−1^. Mice were also administered EdU via the drinking water at 1 mg ml^−1^. Twenty-four hours after the initial injection, the mice were injected with another 10 μg 17β-estradiol plus 1 mg progesterone (Sigma). Forty-eight hours later after the last set of injections, the inguinal mammary glands were dissected out and processed into single cell suspensions as described above and analysed for EdU incorporation using the Click-iT EdU Flow Cytometry Assay Kit (Life Technologies) accordingly to manufacturer’s instructions. All steps of the Click-iT reaction was performed at room temperature (RT). Briefly, inguinal mammary glands were processed to a single cell suspension briefly stained with DAPI, to determine live/dead cells, washed in 1 × PBS and then fixed in Click-iT fixative for 15 min. Cells were washed and permeabilized in 1 × Click-iT saponin-based permeabilization buffer for 15 min. The EdU reaction cocktail (PBS, CuSO_4_, Alexa Fluor 488 azide and buffer additive as per manufacturer’s protocol) was added to the cells for 30 min and then washed in 1% BSA/PBS. Detection of cell-surface antigens (Lineage, CD49f, EpCAM, Sca1 and CD49b, as described above) was then carried out in 1 × Click-iT saponin-based permeabilization buffer. After staining, cells were analysed on a BD LSRII flow cytometry instrument.

### Mammary CFC assay

Single cell suspensions derived from mammary glands were seeded at low density in the presence of an irradiated NIH 3T3 feeder layer in Mouse EpiCult-B (StemCell) media supplemented with 5% fetal bovine serum (StemCell), 10 ng ml^−1^ epidermal growth factor (Sigma), 10 ng ml^−1^ basic fibroblast growth factor (Peprotech), 4 μg ml^−1^ heparin (StemCell) and 50 μg ml^−1^ gentamycin, and were cultured for 7 days at 37 °C in a hypoxic (5% O_2_ and 5% CO_2_) incubator. After 7 days, the cultures were fixed and stained with Giemsa (Raymand A Lab Ltd) and the number of colonies enumerated under a light microscope at × 4 magnification.

### MRU assay

Freshly dissociated donor cells were suspended in 65% PBS supplemented with 25% growth-factor reduced Matrigel (BD) and 10% Trypan Blue solution (0.4%, Sigma) at a concentration such that a 10-μl injection volume contained the desired cell dose. The endogenous mammary epithelium in the inguinal glands of 3-week-old female syngeneic mice was cleared and cells were injected into cleared fat pads as previously described^32^. The mice were mated 3 weeks after surgery and the number 4 glands were removed during pregnancy, and glands fixed and stained as described above. An outgrowth was scored positive if it contained both lobular and ductal elements. Outgrowths were scored in a blind manner. MRU frequencies were calculated using Extreme Limiting Dilution Analysis programme^33^.

### Nucleoside incorporation studies

BrdU, CldU and IdU (all from Sigma) were dissolved in PBS to a concentration of 10 mg ml^−1^ and were injected i.p. or subcutaneously into mice at a dose of 100 mg kg^−1^. For continuous administration of BrdU, mice were given access to water supplemented with 1 mg ml^−1^ BrdU *ad libitum* for up to 56 days. Bottles containing BrdU water were changed every 4 days and were covered with aluminum foil to prevent light exposure.

BrdU-based incorporation into DNA: After required duration of exposure, mice were culled and mammary glands digested as described above. Equal volume of 1 × sterile PBS was injected for the control mice. Flow-sorted cells were spotted onto poly-L-lysine-coated slides and fixed with methanol for 5 min at RT. Cells were then denatured for 30 min at RT with 2 N HCl followed by blocking for 1 h with the M.O.M. Blocking Kit as per the manufacturer’s instructions (Vector Laboratories). Cells were then immunostained with antibodies specific for BrdU (clone Bu20a used at 1 μg ml^−1^, Cell Signaling Technology), CldU (clone BU1/75 used at 1 μg ml^−1^, Abcam) and IdU (clone B44 used at 1 μg ml^−1^, BD Biosciences) according to the manufacturer’s recommendations. BrdU staining was detected using anti-mouse biotin (M.O.M. Blocking Kit) for 30 min at RT followed by 2 μg ml^−1^ streptavidin-AF488 secondary antibody (Invitrogen) for 1 h at RT as per the kit protocol. CldU and IdU co-staining was done using 2 μg ml^−1^ anti-rat AF488 (or AF555), to visualize CldU, and 2 μg ml^−1^ anti-mouse biotin (M.O.M. Blocking Kit) and 2 μg ml^−1^ streptavidin-AF555 (or AF488) secondary antibody (Invitrogen), to visualize IdU. PBS, CldU-injected and IdU-injected mice were used as controls, to ensure specificity of the antibodies. Cells were imaged using Nikon Confocal Upright or Inverted Microscope (Nikon Corporation, USA). Typically, 3,000 cells scored for most cell populations, except when the populations are rare then only 500 cells were scored.

### Calculation of cell division kinetics

To resolve the proliferation kinetics of the different cell populations, we combined two independent strategies based on thymidine incorporation, the first based on continuous BrdU incorporation and the second based on short-term CldU incorporation. Central to our analysis was the observation that, although the oestrus cycle involves short-term intense proliferative activity involving expansion and regression, the long-term behaviour of the tissue is effectively homeostatic, with the cell number being overall conserved in the basal and luminal compartments. From this observation, we can infer that, over the complete oestrus cycle, cell production through proliferation must be perfectly compensated by cell differentiation and loss.

However, to interpret the data on thymidine incorporation, it is important to resolve the lineage relationship of the four putative cell compartments (luminal and basal), as defined in the main text. In particular, the potential transfer of cells between compartments through differentiation could lead to the accumulation of BrdU^+^ cells in one compartment following cell division and BrdU incorporation in another. However, significantly, all four compartments show an initial linear-like increase in the fraction of BrdU^+^ cells, consistent with a pattern of turnover involving the proliferation of independent lineage committed progenitors. (It is noteworthy that the accumulation of BrdU^+^ cells in one compartment solely due to proliferation in another would lead to a nonlinear (*t*^2^) time dependence in the frequency of BrdU^+^ cells in the second compartment.)

Therefore, for a given cell compartment, let us suppose that a fraction, *p*, of the constituent cells are capable of proliferation, whereas the remaining fraction, 1−*p*, are terminally differentiated. Then, to achieve long-term homeostasis (that is, on time scales in excess of the length of the oestrus cycle), cell division must, on average (that is, at the population level), result in asymmetric fate outcome with one cell remaining proliferative and the other committing to terminal differentiation and subsequent loss. If entry into cycle results in just one round of division, for a division rate, 
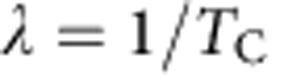
, and loss rate of differentiated cells, 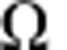, one may show^34^ the fraction of proliferative cells in steady state is given by 
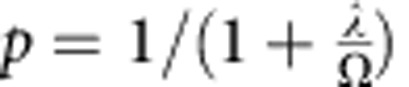
. Then, following continuous BrdU incorporation, the fraction of BrdU^−^ cells is given by,




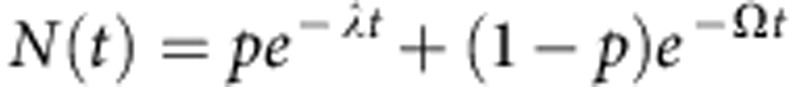




Therefore, for a pure progenitor population (*viz*. 
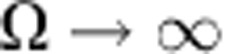
), the fraction decays exponentially, with time constant *T*_C_. Conversely, if turnover relies on a minority progenitor cell population (that is, 
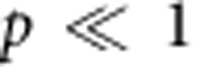
), the fraction is predicted to decay exponentially with time constant, 
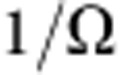
. (More generally, if cells entered an active phase in which they underwent multiple rounds of asymmetric division before re-entry into quiescence, providing the newly born differentiated cells were preferentially lost in the regression phase, the analysis above would be unchanged.)

By contrast, if we consider a short-term CldU pulse, we expect a fraction of




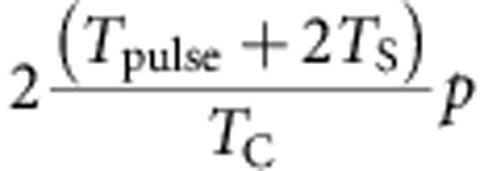




cells to take up CldU, where *T*_pulse_ denotes the length of the pulse and *T*_S_ denotes the length of S-phase, which is estimated to be 7 h (ref. 18). The total number of hours of S-phase where there is exposure to CldU is calculated to be (6+2*T*_S_), as two injections of CldU, with each injection 6 h apart, will capture two S-phase lengths in addition to the 6-h interval. This number of hours is then multiplied by 2, as the analysis following tissue dissociation involves the product of cell division.

Taken together, these two strategies provide two independent measurements from which we can infer the two rates, 
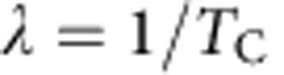
 and 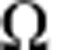. Applying this approach, we initially made the Ansatz that the four cell populations were lineage restricted and were composed predominantly of progenitors. Then, according to the discussion above, the timescale constant (*T*_C_) between consecutive entries into the cell cycle for the different epithelial cell subpopulations was derived from the continuous BrdU incorporation data by making a least-squares fit of the percentage of BrdU^−^ cells lost over time to the exponential decay curve, and using the equation 
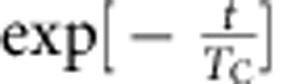
 to determine the respective *T*_C_ values. The validity of assumption was then checked by the analysis of the short-term double-pulse labelling data using the approach above. From the consistency of these results (main text), we confirmed that three of the four cell populations predominantly comprise lineage-restricted progenitors, while the Sca1+ population is heterogeneous, with some 50% of cells are terminally differentiated.

### Immunofluorescence microscopy in tissue sections

Expression of BrdU (clone BU1/75 used at 1 μg ml^−1^, Abcam), CldU (clone BU1/75 used at 1 μg ml^−1^; Abcam), IdU (clone B44 used at 1 μg ml^−1^; BD), Ki67 (clone tec-3 used at a 1:100 dilution, Dako), ER (clone 6F11 used at a 1:100 dilution, Leica Biosystems, or clone D6R2W used at a 1:200 dilution, Cell Signaling Technology), phosphorylated histone H3 (polyclonal used at a 1:500 dilution, Abcam), Aldh1a3 (clone HPA046271 used at a 1:100 dilution, Sigma), CD49b (clone EPR5788 used at a 1:100 dilution, Abcam) and/or K5 (polyclonal used at a 1:1,000 dilution, Abcam) in intact mammary glands was done by fixing freshly isolated glands in 10% neutral buffered formalin overnight and processing the tissue into paraffin. In the phosphorylated histone H3 experiments, mice were injected with BrdU (150 mg kg^−1^ of body weight) 1–24 h before tissue harvesting. Tissue blocks were sectioned at 4 μm, deparaffinized and then heat-induced antigen retrieval in citrate buffer (pH 6). The samples were preblocked in PBS with 1% BSA and 0.1% Tween 20, and then incubated with primary antibodies overnight at 4 °C. The secondary antibodies were goat anti-mouse Cy3 (Jackson Labs), goat anti-rat AF488 and goat anti-rabbit AF647 (Invitrogen), and were all used at 2 μg ml^−1^. No primary antibody was used as a control. Slides were stained with DAPI to visualize the nuclei and sections mounted with ProLong Gold antifade (Invitrogen). In some cases, slides were scanned on the Leica Ariol imaging system using the Olympus BX61 microscope (Leica). Otherwise tissue sections were imaged using the Leica confocal TCS SP5 microscope (Leica).

### 5FU treatment

5FU (Sigma Aldrich) was dissolved in 1 × sterile PBS at a concentration of 10 mg ml^−1^. 5FU was administered by i.p. injection at a concentration of 150 mg kg^−1^ of body weight of the mouse.

### RNA extraction and RT–PCR

Freshly sorted cells were pelleted and the supernatant removed. Total RNA was extracted using the PicoPure RNA extraction kit (Life Technologies) as per the manufacturer’s instructions and samples were treated with DNase using the RNase-free DNase set (Qiagen). Cells from five independent experiments were collected. Total RNA was reverse transcribed into first strand complementary DNA using the Superscript Vilo cDNA Synthesis Kit (Life Technologies). RT–PCR was performed on 1 μl cDNA using an ABI 7900HT Real Time PCR system (Applied Biosystems). Taqman gene expression assay mix was used to detect expression of ERα (Mm00433149_m1, Life Technologies). All raw data was analysed using Sequence Detection System software version 2.4 (Applied Biosystems). The cycle threshold (Ct) values were used to calculate relative RNA expression levels. Expression levels of target gene were normalized to endogenous Gapdh (Mm99999915_g1, Life Technologies) transcripts and compared with the NCL G0/G1 population.

### Telomere length measurements

Mammary epithelial cell subpopulations from 11-week-old female C57/Bl6J mice were sorted as described above. Freshly sorted cells were adhered directly onto poly-L-lysine-coated microscope slides (VWR International) for 30 min at 37 °C. Once adhered, cells were carefully washed twice in PBS and fixed in methanol/acetic acid (3:1 vol/vol), repeated three times for 30 min at RT. Telomere length was then determined as previously described^23^. Briefly, the cells were hybridized with a PNA-tel Cy3-labelled probe, and the DAPI and Cy3 signals were acquired simultaneously into separate channels using a Leica TCS-SP5 confocal ultraspectral microscope and maximum projections from image stacks were generated for image quantification. Quantitative image analysis of telomere fluorescence intensity was performed on confocal images using the Definiens Developer Cell software (Definiens Developer XD). The DAPI image was used to define the nuclear areas that were separated by a Cellenger Solution. After defining the nuclear areas, a predefined Ruleset was used for the quantification of telomere fluorescence intensity (Cy3 image). The fluorescence values for each section were exported to GraphPad Prism and graphs were generated. The total number of telomeric spots scored for each genotype is shown.

### Telomeric repeat amplification protocol

Telomerase activity was measured from fresh flow-sorted mammary epithelial subpopulations using the TRAPeze RT Telomerase Detection kit (Millipore) and previously described^35^. Briefly, samples were lysed in 3-[(3-cholamidopropyl)dimethylammonio]-1-propanesulfonate (CHAPS) lysis buffer and protein concentration determined using the BCA protein assay kit (Pierce). Each telomeric repeat amplification protocol (TRAP) assay included 5 μl of 5 × TRAPeze RT Reaction mix, 0.4 μl (2 units) Hot start Taq Polymerase (New England Biolabs), 17.6 μl nuclease-free water and 2 μl cell extract or control template. The TSR8 control template was used to generate a standard curve according to the manufacturer’s instructions. Reactions were set up in triplicate and the assay was run on the following cycling parameters using a ABI 7900HT Real Time PCR system (Applied Biosystems): 30 min at 30 °C, 2 min at 95 °C, then 45 cycles of 15 s at 94 °C, 60 s at 59 °C and 10 s at 45 °C. Telomerase activity was determined from the equation derived from the linear plot of the log_10_ of the quantities (amoles) of TSR8 control template versus the Ct value of the corresponding concentration of TSR8. The mean values of the quantities for the three replicates of each sample were then calculated. This number was then divided by the amount of protein (ng) contained within the 2 μl cell extract and then divided by 30, to determine the amount of extended telomerase substrate (amoles) produced per ng of protein per minute for each sample.

### Statistical analysis

Data are presented as the mean of independent experiments with s.e.m., unless indicated otherwise. Comparisons between multiple groups were analysed using analysis of variance followed by a multiple comparisons test, as indicated in the figure legends. Statistical significance is indicated as follows: *P<0.05, **P<0.01 and ***P<0.001. MRU frequencies between different cell populations were compared statistically using the Extreme Limiting Dilution Analysis online tool (http://bioinf.wehi.edu.aU/software/elda/)^33^. No statistical method was used to predetermine sample size.

## Author contribution

R.R.G. and M.S. conceived, designed, performed experiments and wrote the manuscript. M.G and M.A.B. performed experiments. B.D.S. and J.S. conceived, designed and wrote the manuscript.

## Additional information

**How to cite this article:** Giraddi, R. R. *et al.* Stem and progenitor cell division kinetics during postnatal mouse mammary gland development. *Nat. Commun.* 6:8487 doi: 10.1038/ncomms9487 (2015).

## Supplementary Material

Supplementary Information Supplementary Figures 1-3, Supplementary Tables 1-5 and Supplementary References

## Figures and Tables

**Table 1:** Cell division during mammary gland development.

**Age (weeks)**	**Absolute number of BrdU**^**+**^ **cells × 10**^**3**^** (% of population)**			
	**Basal**	**Sca1**^−^ **progenitor**	**Sca1**^**+**^ **progenitor**	**NCL**
3	1.9±0.6(19±7)	0.7±0.1(27±5)	0.1±0.1(16±3)	0.4±0.1(25±6)
4.5	2.9±0.9(4±1)	2.8±0.6(15±1)	1.3±0.5(12±4)	1.2±0.7(4±2)
6	1.5±1.0(3±2)	1.9±1.8(5±5)	0.9±0.3(7±1)	7.7±4.5(9±5)
10	4.6±1.9(4±1)	1.3±0.4(4±1)	0.2±0.1(2±1)	9.5±3.6(10±2)

BrdU, 5-bromodeoxyuridine; NCL, non-clonogenic luminal.

Absolute number of different mammary epithelial cell subpopulations that incorporated BrdU in the inguinal mammary glands of 3- to 10-week-old C57Bl6/J mice. Mean±s.e.m of three independent samples for each developmental stage.

**Table 2:** Long- and short-term cell cycle kinetics.

**Population**	**Long-term BrdU** ***T*****c (days)**	**Short-term BrdU** ***T*****c (days)**
Basal	35±4	35±4
Sca1^−^ progenitor	26±3	26±3
Sca1^+^ progenitor	18±3	55±6
NCL	21±3	19±1

BrdU, 5-bromodeoxyuridine; CldU, 5-chloro-2′-deoxyuridine; NCL, non-clonogenic luminal.

*T*c values calculated from long- and short-term nucleoside incorporation studies. *T*c values calculated from the long-term BrdU incorporation studies are derived from pooled data from the 24 independent samples in Fig. 3a, whereas the *T*c values derived from short-term CldU incorporation are derived from eight independent adult mice. Values represent the mean±s.e.m.

**Table 3:** Cycling status of MRUs.

**Oestrus stage**	**Condition**	**Dose**	**Take rate**	**MRU freq (95% CI)**	***P*** **-values**	**Total MRUs per pair glands**
Oestrus	5FU	1,000	12/17	1/617 (1/358–1/1,064)	0.677	5,352
		100	5/13			
	Untreated*	5,000	2/2	1/760 (1/342–1/1,691)		4,550
		2,000	1/1			
		1,000	3/3			
		200	2/15			
Dioestrus	5FU	1,000	7/10	1/642 (1/334–1/1,234)	0.002	4,169
		200	3/4			
		100	1/10			
	Untreated*	5,000	1/1	1/208 (1/138–1/314)		16,702
		2,000	1/1			
		1,000	3/3			
		200	24/39			

5FU, 5-fluorouracil; CI, confidence interval; MRU, mammary repopulating unit.

Sensitivity of MRUs in mammary glands of adult (≥10-week-old) C57Bl6/J mice at different oestrus stages to 5FU (*n*=3–7 donor mice analysed for each oestrus stage and each treatment arm).

^*^Data from untreated mice are extracted from Supplementary Table 5.
