# Supplementary material for: Stem and progenitor cell division kinetics during postnatal mouse mammary gland development
Source: Nat Commun. 2015 Oct 29;6:8487. doi: 10.1038/ncomms9487 (PMC4632194; doi:10.1038/ncomms9487)
Supplement: Supplementary Information — Supplementary Figures 1-3, Supplementary Tables 1-5 and Supplementary References [file ncomms9487-s1.pdf]

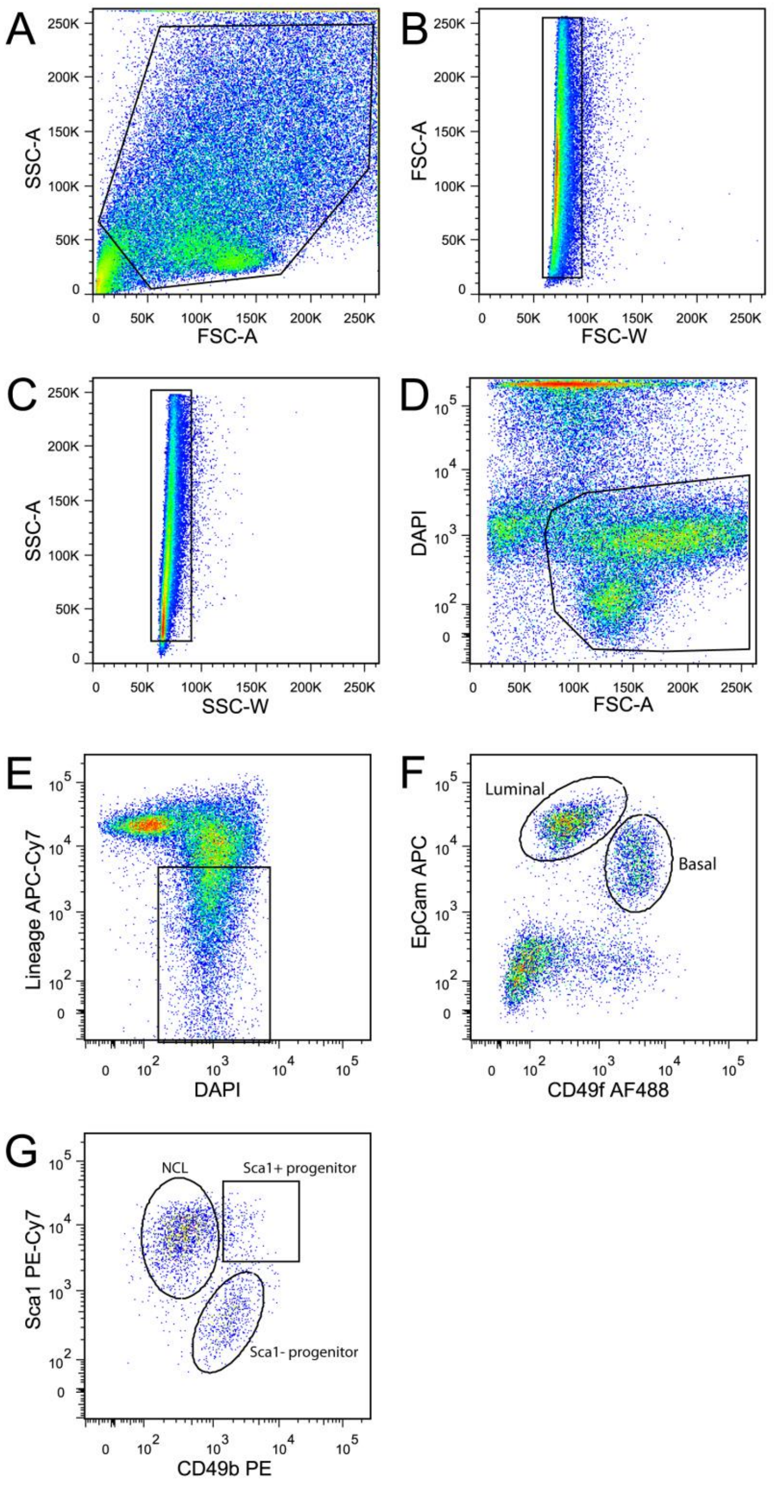

Supplementary Figure 1. Flow cytometry gating. Gating strategy showing the identification and purification of viable basal, Sca1- progenitors, Sca1+ progenitors and NCL cells. The events in panel G are gated on the luminal cell population shown in panel F.

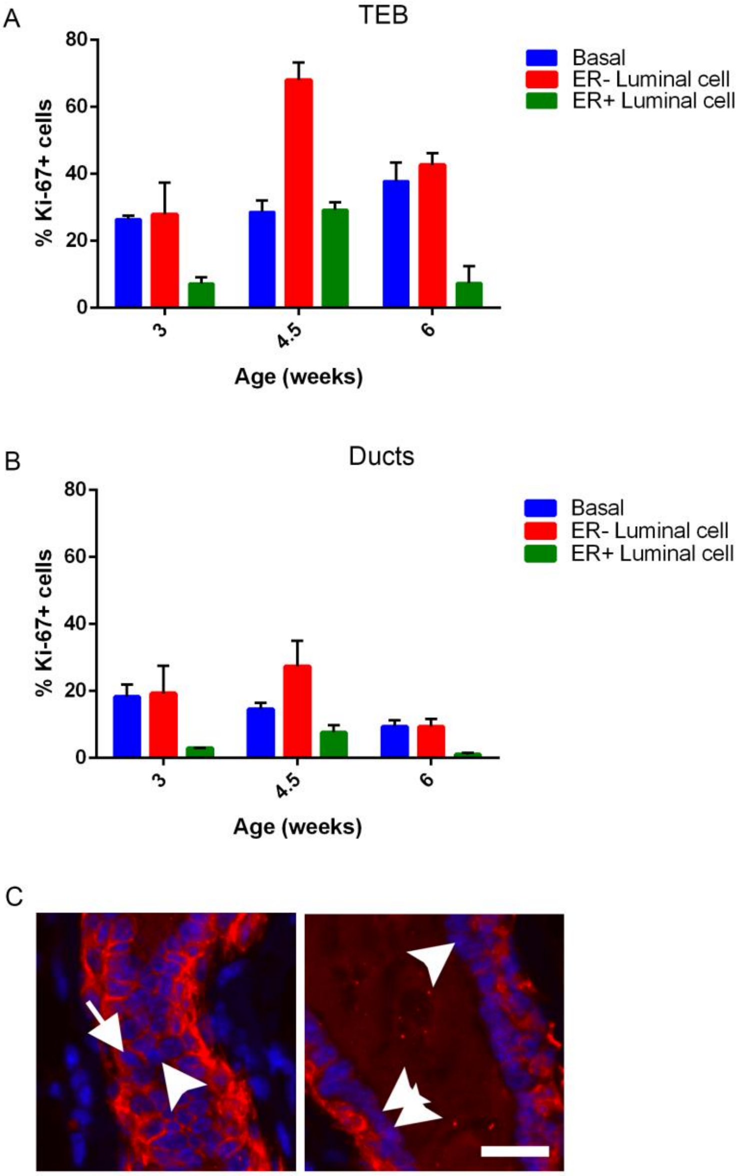

Supplementary Figure 2. Cell division within the mammary epithelium. Quantification data from Figure 2A was extracted and further divided into terminal end buds (A) and ducts (B) and samples recalculated to determine the percentage of Ki-67+ cells within the different developmental stages. (C) Representative zoomed image of CD49b staining from Figure 2D. Arrowheads depict CD49b- NCL cells and arrows indicate CD49b+ progenitors. Scale bar = 20 μm.

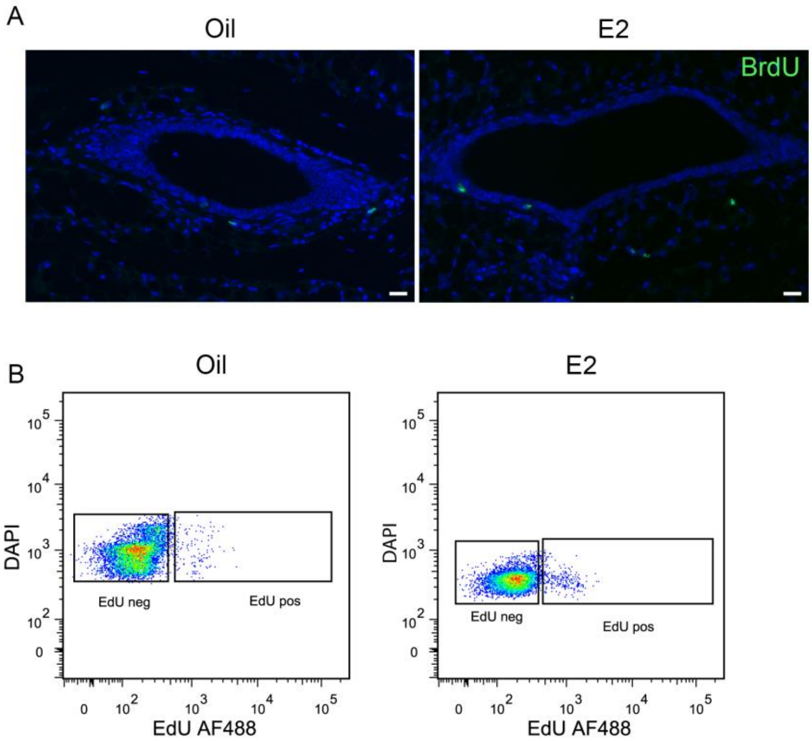

Supplementary Figure 3. Estrogen alone does not induce cell proliferation. Injection of 10 µg of estrogen alone into ovariectomised mice does not induce cell division within the mammary epithelium. Adult (≥10 week-old) mice were ovariectomised, and 7-14 days later, each was injected with either 10 µg of estrogen or oil only, and were administered BrdU or EdU continuously via intraperitoneal injection and the drinking water. Mice were maintained for 8-48 hours, and nucleoside incorporation into the luminal epithelial cells was determined by (A) immunofluorescence microscopy (BrdU is indicated by green fluorescence; scale bar = 20 um) or by (B) flow cytometry. No obvious differences in cell proliferation was observed among the epithelial cells (panel A) or the NCL cells (panel B) above background in oil-treated mice any of the experiments at any timepoints. A total of 6 mice estrogen-treated mice and 2 oil control mice were examined. These results are consistent with those reported in a previous study[1].

Supplementary Table 1

A

Age (weeks)	Basal x 10^3 (% of total epithelium)	Luminal x 10^3 (% of total epithelium)	Sca1$^-$ progenitor x 10^3 (% of total epithelium)	Sca1$^+$ progenitor x 10^3 (% of total epithelium)	NCL x 10^3 (% of total epithelium)
**3**	5.4 ± 14 (76)	1.7 ± 0.3 (24)	0.7 ± 0.2 (10)	0.15 ± 0.07 (2)	0.35 ± 0.17 (5)
**4.5**	38 ± 8 (45)	46 ± 14 (55)	9.6 ± 2.7 (11)	4.2 ± 0.8 (5)	27 ± 10 (32)
**6**	109 ± 13 (59)	77 ± 10 (41)	12 ± 2.5 (6)	5.7 ± 2.1 (3)	36 ± 5 (19)
**10**	131 ± 11 (37)	220 ± 61 (63)	32 ± 11 (9)	18 ± 8.9 (5)	121 ± 31 (34)

B

Age (weeks)	Frequency of MRUs in unsorted cells (95% CI)	Absolute number of MRUs per pair of inguinal glands	Absolute number of Ma-CFCs (x10^2) per pair of inguinal glands
**3**	1 in 22,405 (1/7,358 to 1/68,228)	27	11 ± 0.35
**4.5**	1 in 3,260 (1/1,785 to 1/5,954)	425	37 ± 14
**6**	1 in 926 (1/640 to 1/1,340)	1,403	56 ± 12
**10**	1 in 588 (1/801-1/431)	5,811	300 ± 31

Supplementary Table 1. Cellular content of the mammary gland during development. (A) Absolute number and proportion of different types of cells present in the inguinal mammary glands of 3-10 week-old C57Bl6/J mice (n = 3-4 for each developmental stage). (B) Absolute number of MRUs and Ma-CFCs in the inguinal mammary glands of 3-10 week-old C57Bl6/J (n= 3 for Ma-CFC data, n = 3 to 18 for MRU data). Data is presented as the mean ± s.e.m.

Supplementary Table 2

BrdU exposure time (hours)	% of cells BrdU$^+$ and pH3$^+$	
	Basal	Luminal
**1**	19 ± 1.23	25.6 ± 7.8
**6**	80.7 ± 1.8	86.5 ± 3.9
**12**	20.3 ± 5.3	10.5 ± 3.1
**24**	14.1 ± 3.6	15.0 ± 3.8

Supplementary Table 2. Estimation of the length of S-phase. Mice were administered BrdU once and at different time points between 1 to 24 hours the glands were removed and stained to detect the mitosis specific phospho-histone H3. Maximal dual staining is observed at 6 hours, which represents an approximation of the duration of S-phase. This is similar to the estimate of S-phase for mammalian cells, which has been calculated to be approximately 7 hours[2]. Data is presented as the mean ± s.e.m. from 3 independent mice for each timepoint.

Supplementary Table 3

A

Mouse	BrdU+ Sca1$^-$ progenitor	BrdU+ Sca1$^+$ progenitor	BrdU+ NCL
**1**	713	197	4,812
**2**	2,044	118	7,112
**3**	1,104	310	16,635
**Mean**	1,287	208	9,519
**s.e.m.**	395	56	3,619

B

Mouse	CldU+ Sca1$^-$ progenitor	CldU+ Sca1$^+$ progenitor	CldU+ NCL
**1**	12,867	680	7,630
**2**	7,782	744	6,599
**3**	1,973	241	10,315
**4**	6,204	1,269	22,980
**5**	5,177	773	26,468
**6**	3,267	819	28,706
**7**	5,607	736	39,890
**8**	2,263	720	20,296

Supplementary Table 3. Absolute number of nucleoside$^+$ cells in different mammary cell subpopulations. (A) Data extracted from Figure 2C, but shown for each individual mouse. (B) Number of different types of mammary epithelial cells in 8 different mice that incorporated CldU. Mice were injected twice with CldU, with each injection 6 hours apart and then glands dissociated into a single cell suspension 6 hours after last injection and the different subpopulations isolated by flow cytometry. CldU incorporation was then measured by immunofluorescence microscopy.

Supplementary Table 4

A

Cell subpopulation	Frequency of EdU$^+$ cells (%)	Proportion of all detectable EdU$^+$ cells (%)
Basal	19±5	7±2
Sca1$^-$ progenitor	15±5	17±6
Sca1+ progenitor	29±9	11±2
NCL	13±2	64±7

B

Cell subpopulation	Treatment condition	Frequency of EdU$^+$ cells (%)	Proportion of all detectable EdU$^+$ cells (%)
Basal	Oil control	7.4±0.2	-
	E + P	24±2	27±4
Sca1$^-$ progenitor	Oil control	1.8±0.2	-
	E + P	10±3	4±1
Sca1+ progenitor	Oil control	3.8±1.8	-
	E + P	28±4	2±1
NCL	Oil control	0.8±0.2	-
	E + P	46±6	67±3

Supplementary Table 4. Distribution of EdU$^+$ cells among mammary cell populations. (A) Adult C57Bl6/J mice in proestrus were treated with EdU via the drinking water before culling for analysis at metestrus and the frequency and distribution of EdU$^+$ cells for each epithelial subpopulation was determined. Data presented as the mean ± s.e.m from 5 independent mice. (B) Adult mice were ovariectomised, and two weeks later, each was injected with 10 µg of estrogen and administered EdU via the drinking water. Twenty-four hours later, the mice were injected with 10 µg of estrogen and 1 mg of progesterone and 48 hours later the mice were culled and the frequency of EdU$^+$ cells in the basal, Sca1$^-$ progenitor, Sca1$^+$ progenitor and NCL subpopulations was determined. As well, the distribution of all detectable EdU$^+$ cells in each subpopulation was determined after correcting for population sizes. Mice injected with oil served as controls. Data is presented as the mean ± s.e.m. from 4 independent mice for estrogen and progesterone treatment, and 2 independent mice for oil controls.

Supplementary Table 5

Estrus stage	Dose	Take rate	MRU freq (95% CI)	Total MRUs
Proestrus	5,000	3/3	1/589 (1/272-1/1,275)	7,152
	2,000	3/3		
	1,000	2/2		
	500	2/3		
	200	1/8		
Estrus	5,000	2/2	1/760 (1/342-1/1,691)	4,550
	2,000	1/1		
	1,000	3/3		
	200	2/15		
Metestrus	5,000	9/9	1/1,271 (1/768-1/2,102)	2,288
	2,000	6/9		
	1,000	5/8		
	500	1/3		
	200	2/10		
Diestrus	5,000	1/1	1/208 (1/138-1/314)	16,702
	2,000	1/1		
	1,000	3/3		
	200	24/39		

Supplementary Table 5. MRU numbers during the estrus cycle. The absolute number of MRUs per pair of inguinal glands in adult C57Bl6/J mice in different stages of the estrus cycle was determined by limiting dilution analysis (n = 3-7 donor mice analysed for each estrus stage). Asterisks indicate *** $p<0.001$, ** $p<0.01$, * $p<0.05$ (as determined by using the Extreme Limiting Dilution Analysis online tool (http://bioinf.wehi.edu.aU/software/elda/)).

Supplementary References

1. Beleut, M. et al. Two distinct mechanisms underlie progesterone-induced proliferation in the mammary gland. Proceedings of the National Academy of Sciences 107, 2989-2994, doi:10.1073/pnas.0915148107 (2010).

2. Cameron, I. L. & Greulich, R. C. Evidence for an essentially constant duration of DNA synthesis in renewing epithelia of the adult mouse. J Cell Biol 18, 31-40 (1963).
